# Proteostasis and Diseases of the Motor Unit

**DOI:** 10.3389/fnmol.2016.00164

**Published:** 2016-12-27

**Authors:** Carlo Rinaldi, Imre Mäger, Matthew J. Wood

**Affiliations:** Department of Physiology, Anatomy and Genetics, University of OxfordOxford, UK

**Keywords:** proteostasis, protein quality control, motor unit, intertissue communication, exosomes

## Abstract

The accumulation in neurons of aberrant protein species, the pathological hallmark of many neurodegenerative diseases, results from a global impairment of key cellular processes governing protein synthesis/degradation and repair mechanisms, also known as the proteostasis network (PN). The growing number of connections between dysfunction of this intricate network of pathways and diseases of the motor unit, where both motor neurons and muscle are primarily affected, has provided momentum to investigate the muscle- and motor neuron-specific response to physiological and pathological stressors and to explore the therapeutic opportunities that manipulation of this process may offer. Furthermore, these diseases offer an unparalleled opportunity to deepen our understanding of the molecular mechanisms behind the intertissue communication and transfer of signals of proteostasis. The most compelling aspect of these investigations is their immediate potential for therapeutic impact: targeting muscle to stem degeneration of the motor unit would represent a dramatic paradigm therapeutic shift for treating these devastating diseases. Here we will review the current state of the art of the research on the alterations of the PN in diseases of the motor unit and its potential to result in effective treatments for these devastating neuromuscular disorders.

## The Proteostasis Network

**FIGURE 1:**
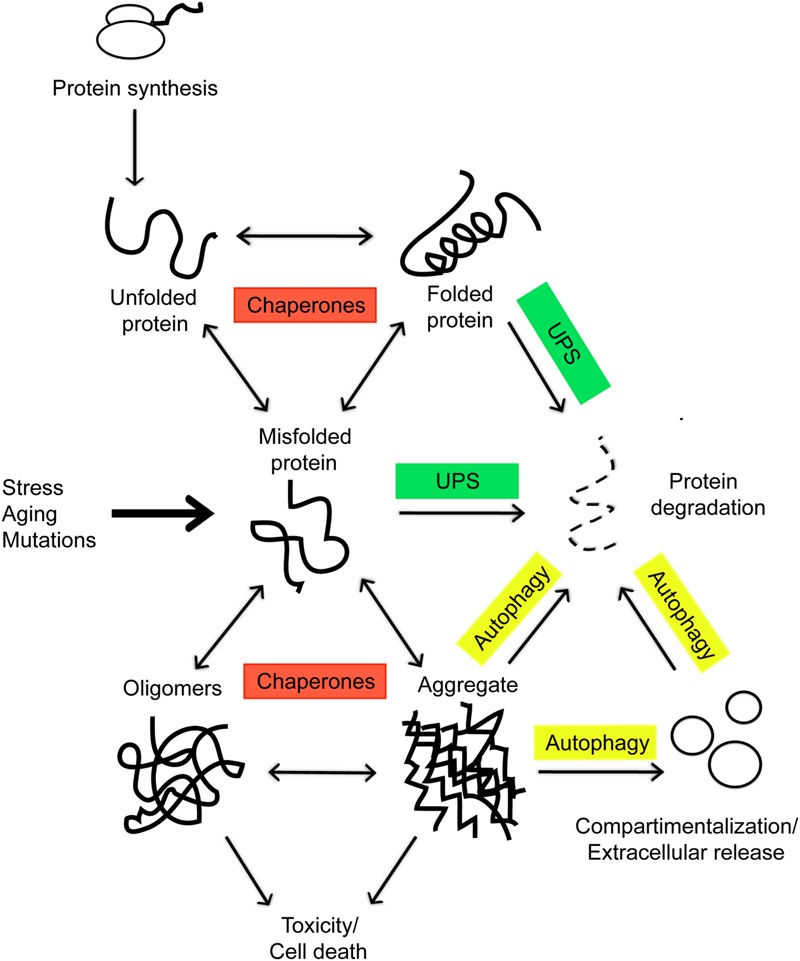
**In eukaryotic cells, as nascent polypeptide chains emerge from ribosomes, they are met by the chaperones and assisted in the folding, which is necessary for a functional protein.** Environmental stress, aging, and mutations can tilt the balance toward formation of misfolded proteins which tend to form toxic aggregates. These incorrectly folded species are detected by a quality-control mechanism and targeted for degradation by the cellular proteolytic systems or secreted via exosomes.

The maintenance of proteome integrity is paramount for cellular and organismal health. Mammalian cells typically express about 20,000 different proteins, which, as they are newly synthesized, must adopt defined three-dimensional configurations to become functional (Balchin et al., 2016). These folded structures are unstable, and proteins, particularly those partially or entirely lacking ordered structure, can misfold under a number of stress conditions or unique metabolic challenges, such as those occurring during cancer or aging, and accumulate in toxic aggregates, the pathological hallmark of many human conditions, including Alzheimer’s and Parkinson’s disease (Balchin et al., 2016). The state of proteome functional balance is commonly referred as proteostasis and the cellular pathways involved in maintaining the integrity of the proteome are collectively referred to as the proteostasis network (PN; Balch et al., 2008). This network coordinates protein synthesis, folding, localization, and turnover and, given its fundamental biological role, requires a tight control within individual cells, tissues, and organs. The main players are chaperones and two proteolytic systems, the ubiquitin-proteasome and the lysosome-autophagy systems, overall comprising almost 1400 components in mammalian cells, which determine whether misfolded proteins will refold into their original stable conformation or whether they will instead be eliminated (Powers et al., 2009) (**Figure 1**). Chaperones are involved in all aspects of proteostasis: they promote the folding of newly synthesized proteins, their translocation across membranes, and the refolding of denatured substrates. Chaperones also prevent aggregation of metastable proteins by targeting misfolded proteins for degradation (Balchin et al., 2016) (**Figure 1**). In eukaryotic cells, two distinct chaperone networks exist: the chaperones linked to protein synthesis (CLIPS; Albanèse et al., 2010), which are linked to the translation machinery and assist folding of newly translated proteins, and the heat shock proteins (HSPs; Treweek et al., 2015), which can be induced by the transcription factor heat shock protein 1 (HSF1) and protect the proteome from stress. Fatally misfolded proteins are polyubiquitinated by an enzymatic E1/E2/E3 ubiquitination cascade and targeted for degradation by the proteasome, the major eukaryotic proteolytic pathway (Goldberg, 2003) (**Figure 1**). Misfolded proteins escaping proteolysis by the ubiquitine-proteasome system (UPS) can reach the lysosome through a membrane transporter in chaperone-mediated autophagy (CMA; Cuervo et al., 2004) or organize into oligomers and/or aggregates, which can be eliminated also by lysosomes through macroautophagy (MA; Jackson and Hewitt, 2016) (**Figure 1**). Numerous studies have highlighted the importance of lysosomal degradation of misfolded proteins in neurodegeneration: knock-down of autophagy genes, such as Atg5 and Atg7, promotes toxicity in mouse models (Hung et al., 2009; Pyo et al., 2013). Conversely, genetic or pharmacologic activation of autophagy has a protective role in a number of neurodegenerative diseases (Nah et al., 2015).

Another mechanism of clearance which is lately gaining increasing interest connected with autophagy is via exosomes, 30–140 nm membrane-bound particles defined by their origin from the endosomal pathway, which are emerging as pivotal players in cellular signaling in both normal physiology and disease states (Baixauli et al., 2014). Recent studies have shown that they can transfer insoluble aggregates to neighboring cells, resulting in a decreased toxic load on the proteome: whether the spreading of protein aggregates via exosomes could provide the molecular basis for the prion-like mechanism of disease propagation in neurodegeneration is certainly very intriguing (Yuyama et al., 2012; Schneider and Simons, 2013; Iguchi et al., 2016) and has the potential to be exploited for therapeutic and biomarker discovery (Boukouris and Mathivanan, 2015; Yuyama et al., 2015).

The PN is highly redundant and all the components are strongly interconnected: impairment of the UPS induces compensatory autophagy, whereas knockdown of autophagy components leads to the accumulation of proteasomal substrates (Korolchuk et al., 2010; Park and Cuervo, 2013).

Additionally, both proteasomal and lysosomal clearance pathways are tightly coupled to the chaperone systems via BAG-domain proteins and specific ubiquitin ligases, such as the cochaperone CHIP (Kabbage and Dickman, 2008). Interestingly, as organisms age, quality control and the cellular response to unfolded protein stress become compromised. Indeed, aging is considered the principal risk factor for the onset of a number of neurodegenerative disorders associated with aggregate deposition (Balchin et al., 2016). Researchers are challenged to develop an integrated view of the PN in health and disease in order to further understand the basic mechanisms of cell function and how loss of proteostasis leads to neurodegeneration.

## Proteostasis and Diseases of the Motor Unit

A motor unit consists of one somatic efferent motor neuron and all of the muscle fibers that it innervates, a concept first introduced by Edward Liddell [1895–1981] and Charles Sherrington [1857–1952] (Duchateau and Enoka, 2011). Because an action potential generated by a motor neuron normally brings to threshold all the muscle fibers it has contact with, a single motor unit neuron constitutes the smallest power unit that can be activated to generate movement.

Many diseases of the motor unit are caused by destabilizing mutations that cause misfolding of the host protein, or alterations in components of the protein homeostasis and quality control machineries, overall suggesting a limited capacity to manage proteome alterations (**Table 1**). Motor neurons are uniquely sensitive to alterations in the PN due to their extreme polarization and post-mitotic nature: they require tight temporal and spatial regulation of protein translation and turnover and cannot rely on the dilution of toxic waste occurring during cell division (Tanaka and Matsuda, 2014). On the other side, systems of protein quality control and in particular fine-tuning the balance between protein synthesis and protein degradation are crucial for the maintenance of muscle mass. Excessive or defective activity of the autophagy-lysosome or ubiquitin-proteasome in acquired or inherited muscle disorders lead to detrimental effects on muscle homeostasis and function (Sandri, 2013). Like in other neurodegenerative diseases, the pathological hallmark of many diseases of the motor unit is the presence of ubiquitin-positive inclusions consisting of misfolded protein aggregates in the affected cells (Hasegawa and Arai, 2007). Several studies have shown that these aggregated structures or, more likely, their oligomeric precursors, directly exert neurotoxic effects by inducing proteotoxic stress and disturbing homeostasis (Ross and Poirier, 2005). Also large aggregates are believed to contribute to the toxicity by sequestering functional proteins and interfering with the UPS and the autophagic degradative pathway (Olzscha et al., 2011). Similarly to alterations in proteostasis in motor neurons, in sporadic and familial inclusion-body myositis (s-IBM), a highly prevalent adult-onset degenerative muscle disease, several proteins, including amyloid-β42 and its oligomers, and phosphorylated tau abnormally accumulate in aggregates, suggesting the presence of similar responses across tissues to proteotoxic insults (Mastaglia and Needham, 2015).

**Table 1:** Diseases of the motor unit associated with mutations in components of the proteostasis network.

Proteostasis system	Gene	Disease	Protein function	Reference
Mutations causing protein misfolding	AR	SBMA	Steroid-hormone activated transcription factor	La Spada et al., 1991
	SOD1	ALS	Enzyme responsible for destroying free superoxide radicals	Rosen et al., 1993
	MYOT	Myofibrillar myopathy	Plays a significant role in the stability of thin filaments during muscle contraction	Salmikangas et al., 1999

Chaperones	DNAJB2	CMT2T, DSMA5	Protein is implicated in protein folding and protein complex dissociation	Blumen et al., 2012
	HSPB8	CMT2L, HMN2A	Small heat-shock protein implicated in macroautophagy	Irobi et al., 2004
	HSPB1	CMT2F, HMN	Involved in stress resistance and actin organization	Ackerley et al., 2006
	DNAJB6	LGMD1D/LGMD1E	Involved in protein folding and oligomeric protein complex assembly	Speer et al., 1999

Ubiquitin-proteasome system	UBQLN2	ALS	Functionally links the ubiquitination machinery to the proteasome	Deng et al., 2011
	VCP	IBMFTD/ALS	Involved in 26S proteasome function, vesicle transport and fusion, and assembly of peroxisomes	Watts et al., 2004

Autophagy	SQSTM1	ALS/FTD	Multi-functional adapter protein that acts as a critical regulator of degradation of ubiquitinated proteins via the proteasome and autophagy	Fecto et al., 2011
	OPTN	ALS/FTD	Can bind to the autophagy receptor SQSTM1 and regulate autophagy flux	Maruyama et al., 2010
	ZFYVE26	SPG15	Involved in the formation and maturation of autophagosomes	Hanein et al., 2008

Vesicle trafficking	RAB7A	CMT2B	Regulates vesicle traffic in the late endosomes and from late endosomes to lysosomes.	Verhoeven et al., 2003
	SIMPLE	CMT1C	Integral membrane protein of lysosome/late endosome	Street et al., 2003
	CHMP2B	ALS/FTD	Component of the heteromeric ESCRT-III complex that functions in the recycling or degradation of cell surface receptors.	Skibinski et al., 2005
	VAPB	ALS/SMAFK	Membrane protein involved in vesicle trafficking	Nishimura et al., 2004

RNA processing	HNRPA1/HNRPA2	IBMFTD/ALS	RNA-binding protein that associates with pre-mRNAs in the nucleus and influence pre-mRNA processing, as well as other aspects of mRNA metabolism and transport	Kim et al., 2013
	TARDBP	FTD/ALS	DNA and RNA-binding protein, which regulates transcription and splicing.	Sreedharan et al., 2008
	FUS	ALS	Protein involved in pre-mRNA splicing and the export of fully processed mRNA to the cytoplasm.	Kwiatkowski et al., 2009
	ATX2	ALS/SCA2	Potentially coordinates an active translation complex	Pulst et al., 1996

*SBMA, spinal and bulbar muscular atrophy; ALS, amyotrophic lateral sclerosis; CMT, Charcot-Marie-Tooth; DSMA, distal spinal and muscular atrophy; HMN, hereditary motor neuropathy; LGMD, limb girdle muscular dystrophy; IBMFTD, inclusion body myositis, fronto-temporal dementia; SPG, spastic paraplegia; SCA, spino-cerebellar ataxia; SMAFK, spinal muscular atrophy, Finkel type.*

Over the past decade, several observations in a group of neurodegenerative diseases have also highlighted important links between protein aggregation and RNA biology (Belzil et al., 2013). A conspicuous number of RNA binding proteins (RBPs) have been shown to be associated with amyotrophic lateral sclerosis (ALS) or other neuromuscular diseases: for example, mutations in RBPs such as hnRNPA1 and A2B1, TDP-43, and FUS are associated with ALS (**Table 1**) (Kabashi et al., 2008; Kim et al., 2013; Qiu et al., 2014). The current prevalent working model suggests that mutations in RBPs enhance dramatically their intrinsic tendency to form stable amyloid structures (Kim et al., 2013), which results in accumulation of ribonucleoproten (RNP) aggregates, disruption of RNA homeostasis or ‘ribostasis,’ and eventually cell death (Ramaswami et al., 2013). Because amyloid initiation is a stochastic event dependent on protein levels, any alteration that increases the local concentration of RBPs in stress granules, such as defects in clearance mechanisms due to VCP mutations, increases its frequency. This may explains why redistribution of the RBP TDP-43 from the nucleus to cytoplasmic inclusions is a hallmark pathological feature of most sporadic and familial forms of ALS and frontotemporal lobar degeneration (FTLD) (Winton et al., 2008). Recent evidence have also highlighted that components of the protein quality control system, such as Hsp70 proteins, survey RNP compositions by influencing mRNA biology (Gilks et al., 2004), suggesting that ribostasis is strongly coupled to the proteostatic state of the cell (Walters and Parker, 2015).

## The Proteostasis Network as Target for Therapy for Diseases of the Motor Unit

The lack of available treatments in the clinic combined with the increasing burden of neurodegenerative disorders in the aging population urgently require the development of effective therapeutic approaches to arrest, stabilize, or reverse the progression of these diseases. As a result of the intrinsic property of the PN to achieve and maintain proteome balance, a single PN modulator has the potential to restore proteostasis in multiple diseases of protein conformations, while both decreasing the levels of the toxic protein and alleviating its pathogenic effects (Balchin et al., 2016). This approach, alone or in synergistic combination with standard single-target therapies (i.e., antisense oligonucleotides), may be more suitable for the complex mechanisms of disease pathogenesis, therefore increasing the chances of success in clinical trials. The use of high throughput screening method to discover molecules able to restore the proteostatic imbalance is rapidly gaining momentum; therapeutic strategies that target the PN are now emerging as promising avenues for the treatment of diseases of protein conformation, based on recent evidence showing that manipulation of the PN in *in vitro* and *in vivo* models are able to suppress the toxicity associated with accumulation of misfolded proteins (Rivas et al., 2015). The ubiquitin proteasome system (UPS) and autophagy have a key role in proteostasis by clearance of proteins and removal of damaged and dysfunctional cellular components. These pathways are essential for normal physiology and development, and are also involved in numerous diseases including cancer and neurodegeneration. Not unexpectedly, these two proteostatic machineries are highly desirable therapeutic targets to improve diseases caused by a mechanism of proteotoxicity (Vidal René et al., 2014; Chondrogianni et al., 2015). Recently, a small-molecule inhibitor of USP14, a deubiquitinating enzyme that inhibits proteasomal degradation by removing the polyubiquitin chain from the client proteins, was shown to enhance degradation of aggregation-prone proteins, suggesting that manipulation of the ubiquitin-proteasome system could be an attractive target to promote the clearance of toxic protein (Lee et al., 2010). The curcumin analog ASC-JM17 ameliorates the disease phenotype in animal models of spinal and bulbar muscular atrophy (SBMA) by targeting the mutant protein, androgen receptor, for degradation via the UPS and simultaneously increasing oxidative stress resistance via activation of the Nrf1 and Nrf2 pathway (Bott et al., 2016). Targeting autophagy may also have therapeutic benefits, as compounds that increase protein turnover by induction of the autophagic activity mitigate neurodegeneration and enhance protein clearance in neuronal models of TDP-43 aggregation (Wang et al., 2012).

Another approach is represented by small molecules that modulate the activity of chaperone proteins, such as Hsp90 and Hsp70, which have essential roles in preventing aberrant protein–protein interactions and promoting correct protein folding (West et al., 2012). The appearance of aggregated proteins, as occurs in diseases of misfolded protein, indicates that the quality control pathways of the PN become compromised, leading to cellular dysfunction and death. Pharmacological agents that increase HSP levels by inducing the heat shock response, such as 17-AAG or arimoclomol have been shown to counteract the toxicity caused by accumulation of toxic proteins in models of SBMA, ALS, and IBM (Kieran et al., 2004; Waza et al., 2005; Ahmed et al., 2016). Interestingly, arimoclomol does not induce the heat shock response in unstressed cells, thereby avoiding the potential side effects associated with widespread heat shock response activation (Kieran et al., 2004; Ahmed et al., 2016). Despite the beneficial effects in preclinical models, several factors need to be considered in order to translate this approach into valuable therapeutic options: the high degree of integration makes manipulation of the PN a challenging task, where the risk of compromising the stability of other proteins will need to be addressed (Balch et al., 2008; Balchin et al., 2016). Also, for conformational diseases involving the motor unit, the proteostasis modulators will need to achieve targeted drug delivery in motor neurons and muscle. Researchers are challenged to develop a better understanding of the complex interplay between molecules, cells, and tissues in order to answer these questions and before moving these strategies into the clinic.

## Intercellular Proteostasis: A Coordinated Response of Tissues and Organs to Proteotoxic Insults

While the central components of protein quality control function autonomously within each cell, it has been recently demonstrated that the PN is also regulated by cell-non-autonomous signaling through communication between subcellular compartments and across different cells and tissues (van Oosten-Hawle et al., 2013). In the evolution from unicellularity to multicellularity, organisms have developed mechanisms of intercellular cooperation in order to protect tissues with intrinsic reduced buffering capacity, such as neurons, and promote a rapid, predictive, and adaptive response to proteotoxic insults (van Oosten-Hawle and Morimoto, 2014).

Most of the discoveries in non-cell-autonomous proteostasis have been made in invertebrates, although evidence of a similar process is starting to emerge also in mammals (Zeineddine and Yerbury, 2015). In *Caenorhabditis elegans*, it has been shown that the heat shock response of somatic cells is regulated by thermosensory neurons that detect temperature changes to control HSF-1 activity throughout the organism (Prahlad et al., 2008). These signaling responses are bidirectional and non-neuronal tissues such as gonad, intestine, or muscle tissue communicate conditions of altered proteostasis with each other and back to the nervous system independently of neural control via autocrine/paracrine and endocrine mechanisms. For example, expression of metastable myosin restricted to muscle cells of *Caenorhabditis elegans* induces a compensatory stress response not only in muscle but also in neuronal and intestinal cells, which relies upon transcriptional feedback regulated by the FoxA transcription factor PHA-4 (van Oosten-Hawle et al., 2013). The motor unit functionally largely relies on anterograde and retrograde signaling at the neuromuscular junction (NMJ): the nature of these signals, particularly from muscle to motor neurons, and the specific molecular mechanisms regulating NMJ structure, function and maintenance are only beginning to be elucidated (Yumoto et al., 2012), mainly due to the technical challenges of studying this complex interaction at the subcellular level. Diseases of the motor unit, such as SBMA and spinal muscular atrophy (SMA), where a mechanism of selective neuronal vulnerability has been challenged by recent evidence showing that therapies exclusively targeting muscle ameliorate the motor neuron pathology and rescue the disease phenotype in animal models (Boyer et al., 2013), represent an ideal working model to unravel the mechanisms of such interaction. Furthermore, the most compelling aspect of these findings is their potential for therapeutic impact: the possibility of manipulating proteostasis in muscle for stemming degeneration of motor neurons, which are less accessible for therapeutic intervention, would represent a dramatic paradigm shift for the treatment of these yet incurable devastating diseases.

**FIGURE 2:**
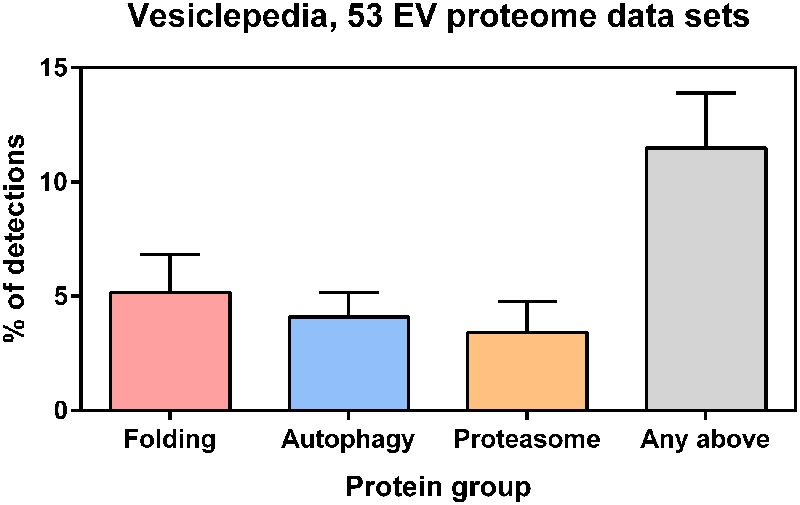
**Protein annotations of specific Gene Ontology (GO) terms were downloaded from the QuickGO database (http://www.ebi.ac.uk/QuickGO/) and cross-referenced with selected EV proteomes from the Vesiclepedia database (http://microvesicles.org, PMID: 23271954).** Only those experiments from Vesiclepedia that described exosome or microvesicle proteomes with >500 detected proteins from *Homo sapiens* or *Mus musculus* samples were included in the analysis. These selection criteria were matched by 53 studies in the Vesiclepedia database where EVs were derived from various cell types (e.g., cell lines, biofluids, primary tumor cells, lymphocytes, myoblasts, platelets, endothelial cells and mesenchymal stem cells). According to the Vesiclepedia database, on average 5.2% of EV proteins are related to protein folding (GO:0006457), 4.1% are related to autophagy (GO:0006914), and 3.4% are related to ubiquitin-dependent proteasomes (GO:0043161). Furthermore, on average 11.5% of EV proteins are related to proteins to any of those processes. Error bars represent standard deviation.

Understanding how the signals of proteostasis are transmitted from one cell to another, how they are integrated in a network and which are the functional implications of such intercellular and intertissue communication in aging and diseases is crucial. In order to boost the organismal proteostasis capacity, it has been recently shown that under conditions of stress, cells can transfer cellular material ranging from stress signals to whole organelles, such as lysosomes, mitochondria, endosomes and lipid droplets through tunneling nanotubes, thin membranous, freely hovering channels with a diameter of 50–200 nm (Astanina et al., 2015; Burtey et al., 2015; Wang et al., 2016). Another mechanism of distant intercellular communication that is currently gaining considerable interest is via extracellular vesicles (Tkach and Théry, 2016). Recent studies suggest that exosomes mediate the non-cell-autonomous control of a number of physiological responses, including organismal proteostasis, by releasing their cargo (RNAs and proteins) to recipient cells and regulating the expression of target genes (Lee et al., 2012). In addition, (i) proteotoxic insults induce the exosome-mediated secretion of HSPs (Takeuchi et al., 2015; Kowal et al., 2016); (ii) adding chaperone-containing exosomes to cultured cells expressing aggregation-prone proteins restores the protein-folding environment in recipient cells (Takeuchi et al., 2015); and (iii) suppression of polyglutamine-mediated neurodegeneration by remote tissue-specific expression of Hsp40 in *Drosophila* depends on Ykt6, an R-SNARE protein necessary for exosome secretion (Takeuchi et al., 2015). Here we show that exosome or microvesicle proteomes derived from various cell types including myoblasts, platelets, endothelial cells and mesenchymal stem cells contain on average of 11.5% proteins related to protein folding, the ubiquitin proteasome, or autophagy (**Figure 2**), suggesting possible PN-related functions of extracellular vesicles as a whole or in their certain subtype. Upon understanding of their biology, it may be possible to use exosomes as vehicle for delivery of therapeutic factors to target tissues (El Andaloussi et al., 2013).

## Concluding Remarks

Given the close ties with proteostasis and diseases of the motor unit, insights into the mechanisms of protein quality control will ultimately impact research striving to decode the pathogenesis of these diseases. In recent years a better understanding of the intracellular systems that govern proteostasis has formed the rationale for numerous successful attempts to target cellular proteostasis to counteract the toxicity related to several neurodegenerative conditions in experimental models. Possible therapeutic approaches include drugs that inhibit toxic aggregate formation, activate protective mechanisms, or promote clearance of toxic aggregates, which might have both prophylactic and therapeutic benefits. Many unresolved issues still remain: understanding why cells occasionally fail to compartmentalize misfolded toxic species, allowing them to interfere with normal protein homeostasis, and how the information of the status of proteostasis is communicated between tissues will be instrumental in elucidating the etiology of these diseases. The possibility that exosomes may function in close relation with autophagy pathway to preserve protein and RNA homeostasis and to mediate the spreading of signals to surrounding cells in order to coordinate organismal systemic responses is intriguing and is a new exciting subject of investigation. Dissecting the complex interplay of proteostasis between different tissues using the motor unit as a working model offers the unparalleled opportunity to gain insight into the general mechanisms of protein quality control and has the potential to provide targets for treatment for these yet incurable conditions.

## Author Contributions

CR: Conception or design of the work, drafting the article, critical revision of the article. IM: Data collection, data analysis, and interpretation, drafting the article. MW: Critical revision of the article, final approval of the version to be published.

## Conflict of Interest Statement

The authors declare that the research was conducted in the absence of any commercial or financial relationships that could be construed as a potential conflict of interest.
